# Efficacy of Mobile App–Based Cognitive Behavioral Therapy for Insomnia: Multicenter, Single-Blind Randomized Clinical Trial

**DOI:** 10.2196/50555

**Published:** 2024-07-26

**Authors:** Jiyoon Shin, Sujin Kim, Jooyoung Lee, Hyerin Gu, Jihye Ahn, Chowon Park, Mincheol Seo, Jeong Eun Jeon, Ha Young Lee, Ji Won Yeom, Sojeong Kim, Yeaseul Yoon, Heon-Jeong Lee, Seog Ju Kim, Yu Jin Lee

**Affiliations:** 1 Department of Psychiatry and Center for Sleep and Chronobiology, Seoul National University Hospital, Seoul National University College of Medicine Seoul Republic of Korea; 2 Department of Psychiatry, Uijeongbu Eulji Medical Center, Eulji University School of Medicine Gyeonggi-do Republic of Korea; 3 Department of Psychiatry, Seoul National University College of Medicine Seoul Republic of Korea; 4 Department of Psychiatry, Samsung Medical Center, Sungkyunkwan University School of Medicine Seoul Republic of Korea; 5 Department of Psychiatry, Severance Hospital Seoul Republic of Korea; 6 Department of Psychiatry, Veteran Health Service Medical Center Seoul Republic of Korea; 7 Chronobiology Institute, Korea University Seoul Republic of Korea; 8 Department of Psychiatry, Korea University College of Medicine Seoul Republic of Korea; 9 Department of Psychology, University of Oregon Eugene, OR United States

**Keywords:** digital therapeutics, mobile app–based cognitive behavioral therapy for insomnia, cognitive behavioral therapy, insomnia, mental health, mobile phone

## Abstract

**Background:**

Cognitive behavioral therapy for insomnia (CBTi) is the first-line therapy for chronic insomnia. Mobile app–based CBTi (MCBTi) can enhance the accessibility of CBTi treatment; however, few studies have evaluated the effectiveness of MCBTi using a multicenter, randomized controlled trial design.

**Objective:**

We aimed to assess the efficacy of Somzz, an MCBTi that provides real-time and tailored feedback to users, through comparison with an active comparator app.

**Methods:**

In our multicenter, single-blind randomized controlled trial study, participants were recruited from 3 university hospitals and randomized into a Somzz group and a sleep hygiene education (SHE) group at a 1:1 ratio. The intervention included 6 sessions for 6 weeks, with follow-up visits over a 4-month period. The Somzz group received audiovisual sleep education, guidance on relaxation therapy, and real-time feedback on sleep behavior. The primary outcome was the Insomnia Severity Index score, and secondary outcomes included sleep diary measures and mental health self-reports. We analyzed the outcomes based on the intention-to-treat principle.

**Results:**

A total of 98 participants were randomized into the Somzz (n=49, 50%) and SHE (n=49, 50%) groups. Insomnia Severity Index scores for the Somzz group were significantly lower at the postintervention time point (9.0 vs 12.8; t_95_=3.85; *F*_2,95_=22.76; η_p_^2^=0.13; *P*<.001) and at the 3-month follow-up visit (11.3 vs 14.7; t_68_=2.61; *F*_2,68_=5.85; η_p_^2^=0.03; *P*=.01) compared to those of the SHE group. The Somzz group maintained their treatment effect at the postintervention time point and follow-ups, with a moderate to large effect size (Cohen *d*=–0.62 to –1.35; *P*<.01 in all cases). Furthermore, the Somzz group showed better sleep efficiency (t_95_=–3.32; *F*_2,91_=69.87; η_p_^2^=0.41; *P*=.001), wake after sleep onset (t_95_=2.55; *F*_2,91_=51.81; η_p_^2^=0.36; *P*=.01), satisfaction (t_95_=–2.05; *F*_2,91_=26.63; η_p_^2^=0.20; *P*=.04) related to sleep, and mental health outcomes, including depression (t_95_=2.11; *F*_2,94_=29.64; η_p_^2^=0.21; *P*=.04) and quality of life (t_95_=–3.13; *F*_2,94_=54.20; η_p_^2^=0.33; *P*=.002), compared to the SHE group after the intervention. The attrition rate in the Somzz group was 12% (6/49).

**Conclusions:**

Somzz outperformed SHE in improving insomnia, mental health, and quality of life. The MCBTi can be a highly accessible, time-efficient, and effective treatment option for chronic insomnia, with high compliance.

**Trial Registration:**

Clinical Research Information Service (CRiS) KCT0007292; https://cris.nih.go.kr/cris/search/detailSearch.do?seq=22214&search_page=L

## Introduction

### Background

Insomnia is a highly prevalent (global prevalence of 2.3%-25.5%) sleep disorder that substantially impacts population health worldwide [1]. Individuals with insomnia often experience decreased quality of life [2] and have a higher risk of developing psychiatric illnesses [3] as well as medical conditions, including cardiometabolic and neurocognitive disorders [4].

Although cognitive behavioral therapy for insomnia (CBTi) is the first-line treatment for insomnia [5-7], its accessibility is limited. These limitations arise due to the time-consuming nature of this treatment and the shortage of trained physicians worldwide [8-10]. Therefore, many individuals face difficulties in obtaining face-to-face CBTi treatment. However, considering the busy working hours, coupled with the high penetration of smartphones in modern society, mobile app–based digital CBTi (MCBTi) may offer easy accessibility to a larger population with minimal space constraints. Digital CBTi is a type of digital therapeutics that uses qualified software programs to deliver evidence-based therapeutic interventions to patients [11] to prevent, manage, or treat medical conditions through effective therapeutic techniques.

Among the various methods of delivering digital CBTi, web-based CBTi, in which a real or web-based therapist provides feedback and supervision, is considered optimal for improving sleep efficiency, sleep onset latency (SOL), and wake after sleep onset (WASO) [12]. Although there have been numerous studies regarding the effectiveness of web-based CBTi [13], the efficacy of MCBTi for improving insomnia and mental health remains unknown [12]. Only a few studies that examined the MCBTi were designed as randomized controlled trials (RCTs) [14] and the control group did not use the same modality (eg, non–app-using control or waitlist control) [15,16]. One MCBTi study with an RCT design used the same modality of control, an active comparator app that provided feedback in chatbot form [10]; the study participants had an Insomnia Severity Index (ISI) score of ≥15, indicating a moderate degree of insomnia. Another multicenter RCT study demonstrated the effectiveness of MCBTi [17], suggesting the inclusion of mental health outcomes in future studies.

In this study, we considered a CBTi mobile app, Somzz, that can be applied to a wider range of insomniacs, ranging from those who experience mild insomnia (ISI score of ≥8) to those with severe insomnia. With this app, CBTi treatment is provided through audiovisual sleep education, guidance on relaxation therapy, and real-time customized feedback on participants’ sleep behavior by analyzing the data entered into the app. Cognitive therapy through a chatbot format is used to correct dysfunctional beliefs related to sleep. In addition, because the effectiveness of sleep restriction has consistently been reported [18], daily *push-alarm* reminders were used to ensure adherence to the prescribed time in bed (TIB).

### Objectives

We aimed to compare the treatment effect of Somzz with an active comparator app providing sleep hygiene education (SHE). We hypothesized that participants using Somzz would demonstrate better outcomes in measures such as the ISI, Epworth Sleepiness Scale (ESS), and sleep diary measures (including sleep efficiency, SOL, WASO, total sleep time, refreshment after sleep, and sleep satisfaction). In addition, we hypothesized that mental health–related issues, including depression, anxiety, and quality of life, would show greater improvement in the Somzz group.

## Methods

### Participants

This multicenter study recruited participants from Seoul National University Hospital, Korea University Anam Hospital, and Samsung Medical Center through advertisements placed in the hospitals and local community (trial registration: KCT0007292). Psychiatrists (H-JL, SJK, and YJL) used the International Classification of Sleep Disorders, Third Edition, to confirm chronic insomnia diagnoses. The participants were recruited between January 2022 and May 2022.

Participants had to meet all of the following inclusion criteria: (1) age of ≥19 years; (2) meeting the diagnostic criteria for chronic insomnia disorder according to the International Classification of Sleep Disorders, Third Edition; (3) ISI score of ≥8; (4) use of Android phones with operating system version 7.0 or above; (5) voluntary decision to participate in the clinical trial and provide written consent on the informed consent form; and (6) willingness to comply with the clinical trial protocol.

Participants were excluded from the study if any of the following exclusion criteria were met: (1) presence of sleep disorders other than chronic insomnia disorder (eg, hypersomnia, narcolepsy, or sleep-disordered breathing); (2) presence of serious medical illnesses; (3) underlying disorders that could be exacerbated by sleep restriction (eg, bipolar disorder and psychosis); (4) severe depression, defined by a Hamilton Depression Rating Scale score of ≥24; (5) presence of medications (eg, chronic use of stimulant medication) or lifestyle factors that provoke insomnia (eg, caffeine, alcohol, or tobacco addiction); (6) challenging schedules that make it difficult to receive sleep interventions (eg, night shift workers or rotating shift workers); (7) presence of auditory or cognitive impairments that make it difficult to undergo treatment using the investigational medical device for the clinical trial; (8) difficulty using universal devices such as smartphones; (9) lack of completion of electronic sleep diaries on at least 7 occasions within 10 days from the first day of electronic sleep diary entry; (10) being pregnant or lactating; (11) current participation in another clinical trial or participation in another clinical trial within 90 days from the screening date; and (12) in the judgment of the investigator, being deemed inappropriate for participation in the clinical trial due to the ethical or potential impact on the clinical trial results.

Individuals who met the study inclusion criteria were randomly assigned to either the Somzz or SHE group at a 1:1 ratio. In this single-blind RCT, the participants were blinded to group allocation. Random allocation was performed using an interactive web response system and a predetermined randomization list. Stratified block randomization was implemented by statisticians using the SAS PROC PLAN procedure; the block size was predetermined for each clinical trial site. Stratification was conducted based on the recruiting institution. The block size and seed number used were selected randomly by the responsible statistician for randomization.

The intervention and control groups had the same visit schedule, and visits were supervised by clinicians or trained psychologists. Written consent was obtained before clinical assessments (eg, vital signs, physical examination, assessment of combination therapy, completion of self-reported scales, and System Usability Scale assessment). However, all therapeutic interventions for insomnia were exclusively administered through the Somzz app. There were 6 visits in total: visit 1 involved screening for study eligibility; visit 2 (week 0) encompassed the collection of baseline demographic and ISI data; visits 4 (week 2) and 6 (posttreatment time point) involved assessment of treatment adherence and completion of sleep-related, mental health, and quality of life scales; and visits 3 (week 1) and 5 (week 4)—both telephone visits—consisted of ISI and ESS completion.

### Ethical Considerations

The study protocol was approved by the Institutional Review Board for Human Subjects of Seoul National University Hospital (2109-145-1258). Detailed information about the study was provided to the participants, and written informed consent was obtained before enrollment. A transportation subsidy of 50,000 KRW (US $42) was provided for each of the four visits to the institution. The collected data were anonymized.

### Sample Size Calculation

Sample size was calculated based on group differences and SDs reported in previous papers regarding digital CBTi (the Food and Drug Administration–approved “SHUT-I” program) [19,20]. Assuming an intergroup difference of –4.6 (SD 6.2), a significance level of 5%, and a power of 90%, the sample size calculation yielded 39 participants per group. Assuming a dropout rate of 20%, we intended to recruit 49 participants per group—98 participants in total.

### Intervention

**Figure 1:**
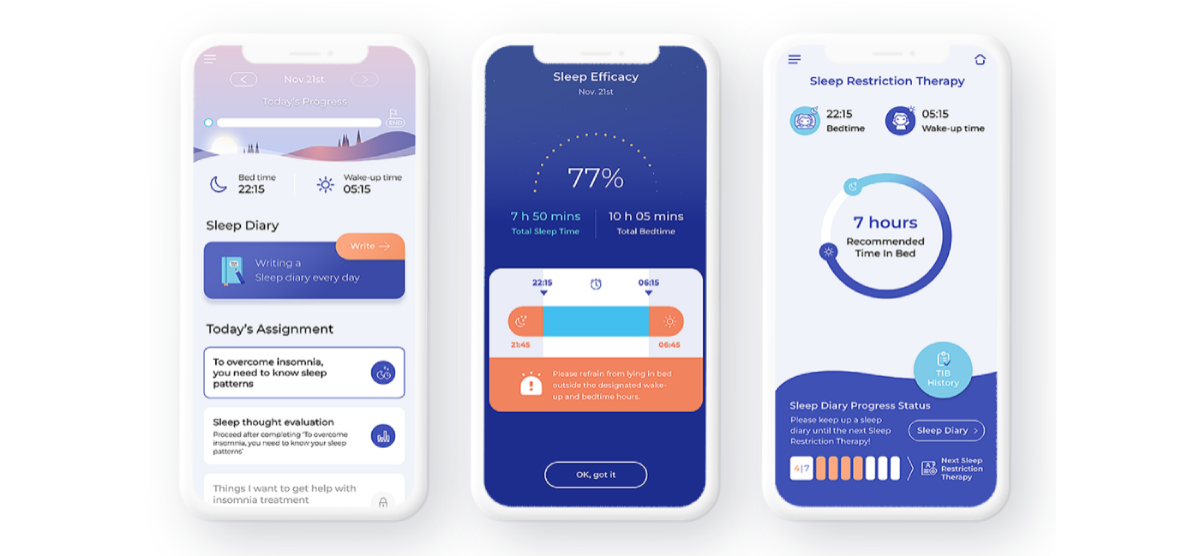
Example screenshots of the Somzz app.

Somzz is a mobile app software medical device designed for the treatment of chronic insomnia. It implements the CBTi protocol, incorporating stimulus control; sleep restriction; SHE; and cognitive therapy, the first-line treatment for chronic insomnia. The app consists of six steps conveyed for 6 weeks: (1) general sleep education, (2) stimulus control and sleep restriction, (3) SHE, (4) relaxation techniques, (5) cognitive therapy, and (6) relapse prevention and termination (Table 1). The Somzz group participants were provided with real-time data-based customized feedback, daily assignments, behavioral interventions (eg, recommendations for TIB), and push notification messages (eg, when to write daily in the sleep diary and when to go to bed considering past TIB) during the 6-week intervention. The intervention was conducted in a fully automated manner. Screenshots of Somzz are presented in Figure 1.

Real-time TIB recommendations constitute a primary feature of Somzz. On the basis of each participant’s sleep schedule as recorded in their sleep diary, the app calculates their sleep efficiency and prompts them to adjust their TIB as necessary. If a participant stays in bed longer than the recommended time, real-time feedback is provided in the form of alarms. In addition, as part of a daily *to-do* list, push notifications encourage participants to complete >6000 steps during the day and ensure sufficient exposure to sunlight. Somzz also incorporates a chatbot module, allowing users to change any dysfunctional beliefs related to insomnia by answering the chatbot therapist’s questions. The same user interface was used for SHE, although the sleep diary module was only 6 weeks in duration and no TIB recommendations were provided. These differences in features may have led to differences in long-term treatment outcomes between the Somzz and SHE groups, as revealed by the linear mixed model analysis of ISI scores, ESS scores, and sleep efficiency.

The SHE group participants also installed the active comparator app on their phones, which comprised SHE and a daily sleep diary. However, no push notification messages or TIB prescriptions were sent to the participants. Sleep education in both apps were provided through audiovisual materials (eg, movie clips including narrations and text).

Participants were followed for 6 weeks during the intervention, and those who consented to extended follow-ups were evaluated for an additional 4 months after the intervention (with 4 additional visits per month) to assess the maintenance of treatment effects.

**Table 1:** Contents of the Somzz sessions.

Session	Contents	Additions
Session 1: basic education	When a patient first signs up on the app, they undergo a sleep assessment to evaluate their basic information, the severity of insomnia symptoms, and the presence of comorbid conditions. In addition, they receive basic education and set goals.	Homework
Session 2: stimulus control and sleep restriction	Somzz provides education on stimulus control, behavioral interventions, and feedback. The goal is to use the bed or bedroom solely for sleeping purposes and to engage in sleep-disruptive activities outside the bedroom. This aims to regulate sleep by adjusting the sleep environment, bedtime, and bedroom conditions in relation to sleep. It is a behavior therapy technique that associates healthy habits conducive to falling asleep with modifying maladaptive conditioning related to sleep. Avoiding activities such as watching television, work-related behaviors, and using the internet or mobile phones in bed is encouraged, and daily compliance is recorded and reviewed during CBTi^a^ sessions. Somzz also provides education on sleep restriction, behavioral interventions, and feedback. In other words, we assess the patient’s sleep duration through a sleep diary to implement sleep restriction. Considering the patient’s sleep efficiency, we determine the target sleep duration and wake-up time. We gradually adjust the total TIB^b^ by 15 minutes per week until reaching the target sleep efficiency of 85%.	Homework, feedback, and TIB recommendations
Session 3: sleep hygiene education	Somzz provides education on sleep hygiene, behavioral interventions, and feedback. This involves examining factors that can impact sleep, such as excessive consumption of coffee or alcohol, and providing education to establish healthy habits for sleep. Subsequently, we encourage daily recording of sleep habits and provide feedback on them.	Homework, feedback, and TIB recommendations
Session 4: relaxation therapy	Somzz provides education on relaxation therapy, behavioral interventions, and feedback. This includes training on relaxation techniques such as diaphragmatic breathing and progressive muscle relaxation, providing a structured self-training schedule, and offering feedback on performance.	Homework, feedback, and TIB recommendations
Session 5: cognitive therapy	Somzz provides cognitive therapy counseling and feedback. This therapy aims to address and correct automatic thoughts related to insomnia that contribute to worsening sleep. It focuses on examining and correcting dysfunctional thoughts associated with excessive worry about insomnia, anxiety arising from perceived health and psychological effects of insomnia, and thoughts characterized by excessive preoccupation with sleep duration. The therapy also targets catastrophic thinking regarding the consequences of poor sleep and generalizations attributing discomforting symptoms solely to sleep-related causes.	Homework, feedback, and TIB recommendations
Session 6: relapse prevention and termination	Somzz conducts a reassessment of symptoms before program termination and provides feedback on progress compared to the initial evaluation. We also provide feedback and conduct a relapse prevention education session followed by a final evaluation.	Homework and feedback

^a^CBTi: cognitive behavioral therapy for insomnia.

^b^TIB: time in bed.

### Outcomes and Measures

The primary outcome was the comparison of total ISI score between the Somzz and SHE groups at each clinical and postintervention follow-up visit [21]. Secondary outcomes included sleep diary and psychiatric measures. Sleep diary measures recorded via the Somzz app included SOL, sleep efficiency, total sleep time, number of awakenings during sleep, WASO, refreshment after waking up, and satisfaction with sleep. Psychiatric measures included daytime sleepiness, as indicated using the ESS [22]; dysfunctional beliefs, measured using the Dysfunctional Beliefs and Attitudes About Sleep Scale (DBAS) [23]; depressive symptom severity, measured using the Patient Health Questionnaire–9 (PHQ-9) [24]; anxiety symptom severity, measured using the Generalized Anxiety Disorder Scale–7 [25]; fatigue, measured using the Fatigue Severity Scale (FSS) [26]; and health-related quality of life, measured using the 36-item Short Form Health Survey (SF-36) [27] and the EQ-5D-5L [28].

ISI and ESS scores were obtained at each visit, and sleep diary measures were evaluated daily. Other self-report scales, including psychiatric, fatigue, and quality of life measures, were completed at baseline, the postintervention time point, and during the 4-month follow-up period. We defined remission as an ISI score of <8 after treatment; thus, a decrease in the ISI score of >7 points from baseline was defined as a treatment response.

### Statistical Analysis

ISI scores, sleep diary, and psychiatric measures were compared between the 2 groups using an analysis of covariance adjusting for baseline severity and within groups using a paired 2-tailed *t* test. Regarding the calculation of effect sizes in the paired *t* test, in the following equation, *s_p_* is the pooled *s_d_*, *n* is the number of paired observations, *X_i_* represents the difference in each paired observation, and is the mean of these differences:



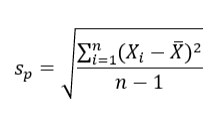



**(1)**

Linear mixed model analysis was conducted to examine the group-time interaction regarding ISI and ESS scores and sleep diary measures. Factors associated with remission (ISI score of <8) and response (ISI score decrease from baseline of >7) were evaluated using binary logistic regression. The primary and secondary outcomes were analyzed using the intention-to-treat principle; the analysis included all participants randomly allocated to either the Somzz or SHE group. In addition, the last observation carried forward method was used to handle missing data. All analyses were conducted using R (version 4.2.0; The R Foundation). We used the R package *lme4* [29] to fit the linear mixed model. Prespecified analysis plan can be found in Multimedia Appendix 1 [30, 31 ].

## Results

### Overview

**Figure 2:**
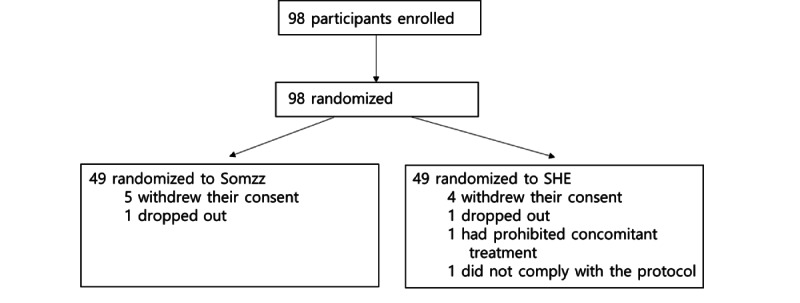
Flowchart of participant enrollment. SHE: sleep hygiene education.

A total of 98 participants were randomly assigned to the Somzz and SHE groups, with each group consisting of 49 (50%) individuals. Of these 98 participants, 5 (5%) individuals from the Somzz group and 4 (4%) from the SHE group withdrew their consent, and 1 (1%) participant in each group did not complete the study (Figure 2). The baseline demographic data are presented in Table 2. In total, 71 participants (34/49, 69% in the Somzz group and 37/49, 76% in the SHE group) consented to participate in the extended (4-month) follow-up study.

**Table 2:** Baseline demographic data for the Somzz and sleep hygiene education (SHE) groups.

				Somzz (n=49)		SHE (n=49)
**Sex, n (%)**						
	Male			20 (41)		18 (37)
	Female			29 (59)		31 (63)
Age (years), mean (SD)				44.1 (13.1)		40.5 (12.8)
**Insomnia duration, n (%)**						
	3-6 months			2 (4)		4 (8)
	6-12 months			4 (8)		5 (10)
	1-3 years			17 (35)		12 (24)
	3-5 years			7 (14)		12 (24)
	Approximately 5 years			19 (39)		16 (33)
**Occupation, n (%)**						
	Employed full-time			13 (27)		20 (41)
	Employed part-time			9 (18)		8 (16)
	Retired			1 (2)		2 (4)
	Student			4 (8)		6 (12)
	Unemployed			14 (29)		8 (16)
	Other			8 (16)		5 (10)
Current medical illness, n (%)				17 (35)		17 (35)
**Current use of hypnotics, n (%)**				9 (18)		9 (18)
	Days of use per week, mean (SD)			3.9 (2.5)		4.2 (2.6)
**Caffeine use**						
	**Current user, n (%)**			35 (71)		36 (73)
		Caffeine units, mean (SD)	1.7 (1.1)		1.5 (1.0)	
	Ex-user, n (%)			2 (4)		1 (2)
	Never, n (%)			12 (24)		12 (24)
**Smoking**						
	**Current smoker, n (%)**			9 (18)		4 (8)
		Smoking units, mean (SD)	4.8 (4.8)		3.5 (2.4)	
		Duration of use (years), mean (SD)	9.9 (10.0)		11.8 (7.7)	
	Ex-smoker, n (%)			4 (8)		8 (16)
	Never smoker, n (%)			36 (73)		37 (76)
**Drinking**						
	**Current drinker, n (%)**			26 (53)		28 (57)
		Drinking units, mean (SD)	2.5 (3.0)		3.5 (4.5)	
		Duration of use (years), mean (SD)	19.8 (12.5)		14.9 (11.4)	
	Ex-drinker, n (%)			1 (2)		4 (8)
	Never drinker, n (%)			22 (45)		17 (35)
**Psychiatric diagnosis, n (%)**				5 (10)^a^		6 (12)
	Depressive disorder			4 (8)		4 (8)
	Anxiety disorder			1 (2)		—^b^
	Panic disorder			1 (2)		1 (2)
	Bipolar II disorder			1 (2)		1 (2)
	Alcohol use disorder			1 (2)		—

^a^One participant had depression, panic disorder, and an alcohol use disorder, and another one was diagnosed with depression and anxiety.

^b^Not applicable.

### Primary Outcomes

**Figure 3:**
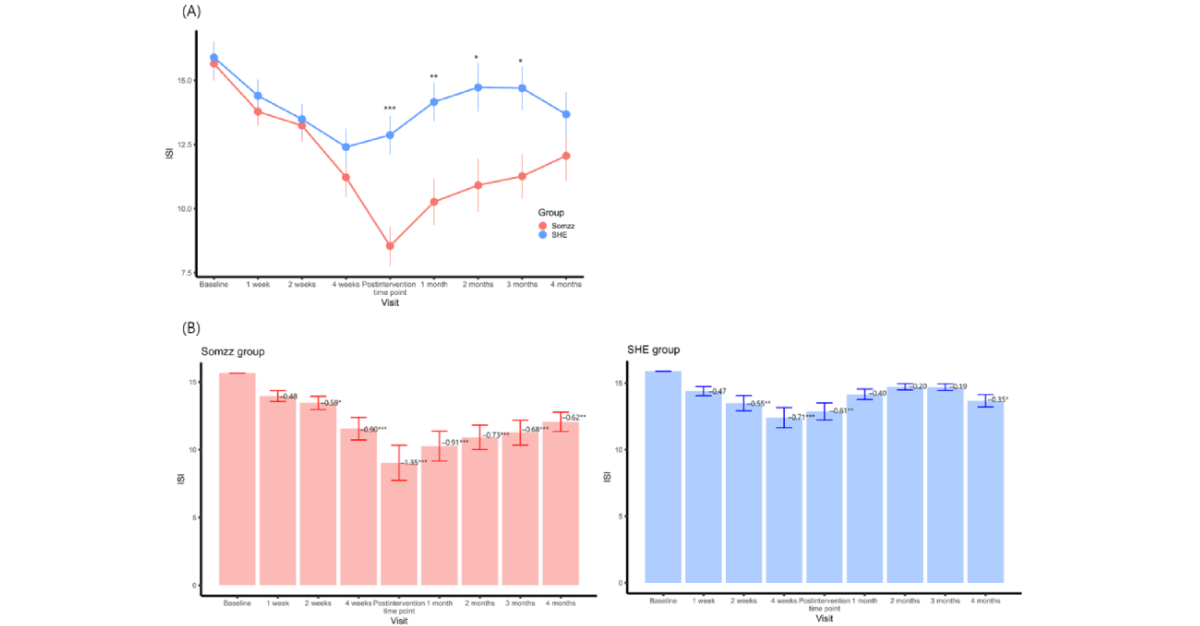
Insomnia Severity Index (ISI) scores at each time point. (A) Between-group comparison of ISI scores at each time point (analysis of covariance). (B) Within-group analysis of ISI scores at each clinical visit compared with baseline scores (2-tailed paired t test). The numbers on the bar graphs are Cohen d values. Error bars are also presented. In the Somzz and sleep hygiene education (SHE) groups, 69% (34/49) and 76% (37/49) of the participants, respectively, consented to take part in the 4-month follow-up. The postintervention time point was 6 to 9 weeks after the baseline. *P<.05; **P<.01; ***P<.001.

Comparison of the ISI scores between the Somzz and SHE groups showed that the scores of the Somzz group were lower at the postintervention time point (9.0 vs 12.8; t_95_=3.85; *F*_2,95_=22.76; η_p_^2^=0.13; *P*<.001) and at the 1-month (10.3 vs 14.2; t_68_=3.24; *F*_2,68_=18.63; η_p_^2^=0.14; *P*=.002), 2-month (10.9 vs 14.7; t_68_=2.51; *F*_2,68_=10.94; η_p_^2^=0.09; *P*=.02), and 3-month (11.3 vs 14.7; t_68_=2.61; *F*_2,68_=5.85; η_p_^2^=0.03; *P*=.01) follow-ups (Figure 3A). At the 4-month follow-up, no difference in ISI scores was found between the 2 groups (12.1 vs 13.7; t_68_=0.95; *F*_2,68_=6.19; η_p_^2^=0.07; *P*=.35). The linear mixed model analysis with a random slope revealed a significant group-time interaction effect from baseline to the postintervention time point (estimate=0.76; t_96_=3.02; marginal *R*^2^=0.55; *P*=.003) and from the postintervention time point to the 4-month follow-up (estimated=–0.72; t_70.25_=–2.64; marginal *R*^2^=0.47; *P*=.01). However, when considering baseline to the 4-month follow-up, the model was not significant (estimate=0.32; t_79.61_=1.94; marginal *R*^2^=0.45; *P*=.06).

The Somzz group showed treatment effects on the ISI score from intervention week 2 (Cohen *d*=–0.59; *P*=.02); the effect was sustained up to the 4-month follow-up (Cohen *d*=–0.62; *P*=.003) compared to the baseline ISI score (Figure 3B). The maximum treatment effect was observed at the postintervention time point (Cohen *d*=–1.35; *P*<.001). The SHE group also showed improvement at intervention week 2 (Cohen *d*=–0.55; *P*.004), week 4 (Cohen *d*=–0.71; *P*<.001), the postintervention time point (Cohen *d*=–0.61; *P*=.002), and the 4-month follow-up (Cohen *d*=–0.35; *P*=.04; Figure 3B).

Both the Somzz and SHE groups showed improvements in ISI scores compared to baseline. In the Somzz group, the maximum improvement in ISI score was observed at the postintervention time point (Cohen *d*=–1.35), and the intervention effect decreased gradually during the follow-up period (Figure 3B). The treatment effect was maintained at the 4-month follow-up in the Somzz group (Cohen *d*=–0.62; Figure 3B). However, there was no difference between the groups at that point (estimate=1.16; t_68_=0.95; *F*_2,68_=6.19, η_p_^2^=0.07; *P*=.35; Figure 3A).

The proportion of individuals in remission at the postintervention time point was 45% (22/49) in the Somzz group, whereas in the SHE group, the proportion was 12% (6/49; risk ratio=3.67, 95% CI 1.32-10.20; *P*.01), and the risk difference was 0.33 (95% CI 0.11-0.54; *P*.002). Regarding the intervention response, there were 57% (28/49) of responders in the Somzz group and 22% (11/49) of responders in the SHE group (risk ratio=2.55, 95% CI 1.06-6.12; *P*.04), and the risk difference was 0.35 (95% CI 0.10-0.60; *P*.006).

### Sleep Diary Measures

**Figure 4:**
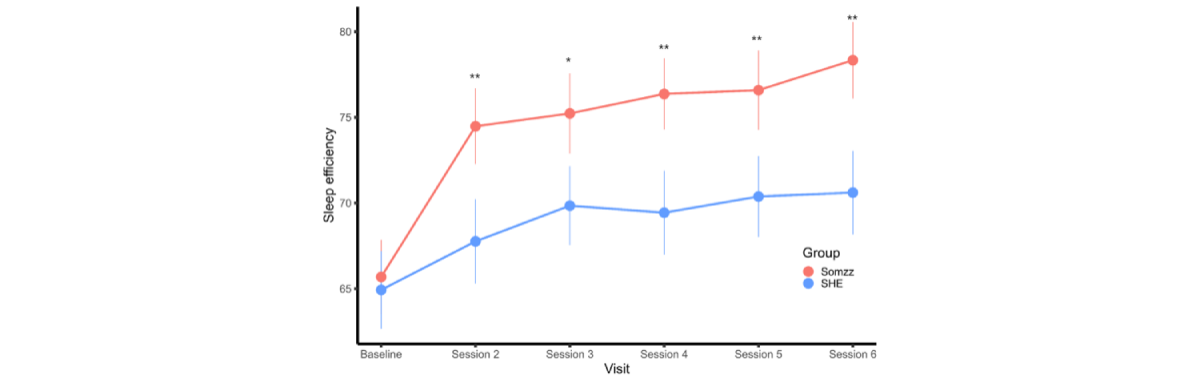
Comparison of sleep efficiency via sleep diary measures between the Somzz and sleep hygiene education (SHE) groups. Group comparison of Insomnia Severity Index scores at each time point using analysis of covariance adjusting for the baseline score. *P<.05; **P<.01; ***P<.001.

The sleep diary measures were compared between the 2 groups with adjustment for baseline measures (Multimedia Appendix 2). Significant group differences were observed in sleep efficiency during treatment sessions 2 to 6 (Multimedia Appendix 2 and Figure 4). The Somzz group also had a shorter WASO and higher satisfaction levels after sleep compared to the SHE group after the intervention.

The linear mixed model analysis with a random slope revealed significant group-time interaction effects on sleep efficiency in the treatment sessions (estimate=–1.00; t_92_=–2.70; adjusted *R*^2^=0.71; *P*=.008).

### Self-Report Questionnaires

With adjustment for baseline severity, scores for self-report measures including the ESS, DBAS, PHQ-9, FSS, and SF-36 significantly differed between the Somzz and SHE groups after the intervention (Table 3). In particular, the differences in DBAS and FSS scores persisted at the 4-month follow-up (Table 3).

The linear mixed model analysis with a random slope revealed a significant group-time interaction effect on ESS from baseline to the postintervention time point (estimate=0.59; t_90.90_=3.48; marginal *R*^2^=0.51; *P*<.001), from the postintervention time point to the 4-month follow-up (estimate=–0.49; t_300.56_=–2.58; marginal *R*^2^=0.43; *P*=.01), and from baseline to the 4-month follow-up (estimate=0.28; t_79.11_=3.35; marginal *R*^2^=0.49; *P*=.001).

**Table 3:** Comparisons of self-report questionnaires between the Somzz and sleep hygiene education (SHE) groups^a^.

			Somzz (n=49)					SHE (n=49)					ANCOVA^b^ statistics						
			Values, mean (SD)		Cohen *d*		Values, mean (SD)			Cohen *d*		*t* test (*df*)			*F* test (*df*)		η_p_^2^		*P* value
**ESS^c^**																			
	Baseline	6.7 (3.9)		—^d^		6.4 (3.4)			—		—			—		—		—	
	Postintervention time point	4.3 (3.2)		–0.72^e^		6.6 (4.3)			0.01		3.82 (95)			32.38 (2, 94)		0.22		<.001	
	4-month follow-up	6.1 (3.7)		–0.51		5.8 (3.6)			–0.17		0.84 (68)			26.10 (2, 67)		0.28		.41	
**DBAS^f^**																			
	Baseline	107.3 (21.2)		—		99.8 (25.8)			—		—			—		—		—	
	Postintervention time point	74.0 (33.7)		–1.01^g^		98.4 (20.3)			–0.10		5.32 (95)			21.55 (2, 94)		0.10		<.001	
	4-month follow-up	80.2 (28.5)		–1.06^g^		94.9 (22.8)			–0.17		3.35 (68)			12.97 (2, 67)		0.13		.001	
**PHQ-9^h^**																			
	Baseline	9.4 (4.9)		—		10.4 (5.0)			—		—			—		—		—	
	Postintervention time point	6.6 (5.2)		–0.67^e^		8.7 (3.9)			–0.36		2.11 (95)			29.64 (2, 94)		0.21		.04	
	4-month follow-up	6.8 (4.6)		–0.62^i^		8.6 (4.8)			–0.34		1.21 (68)			9.90 (2, 67)		0.11		.23	
**GAD-7^j^**																			
	Baseline	6.6 (4.5)		—		7.6 (4.6)			—		—			—		—		—	
	Postintervention time point	4.9 (4.5)		–0.44		6.7 (3.7)			–0.27		1.69 (95)			35.36 (2, 94)		0.25		.10	
	4-month follow-up	5.4 (4.6)		–0.28		6.3 (4.5)			–0.38		0.35 (68)			6.96 (2, 67)		0.09		.73	
**FSS^k^**																			
	Baseline	39.8 (12.8)		—		44.8 (8.9)			—		—			—		—		—	
	Postintervention time point	33.6 (13.8)		–0.71^i^		44.5 (9.0)			–0.13		3.88 (95)			87.91 (2, 94)		0.41		<.001	
	4-month follow-up	34.5 (12.6)		–0.60		42.1 (10.0)			–0.39		2.04 (68)			41.62 (2, 67)		0.33		.046	
**SF-36^l^**																			
	Baseline	65.4 (16.9)		—		62.5 (15.2)			—		—			—		—		—	
	Postintervention time point	72.4 (16.8)		0.58^i^		63.5 (14.1)			0.08		–3.13 (95)			54.20 (2, 94)		0.33		.002	
	4-month follow-up	68.9 (20.8)		0.21		66.0 (14.3)			0.19		–0.59 (68)			12.07 (2, 67)		0.15		.56	
**EQTOTAL^m^**																			
	Baseline	7.6 (2.0)		—		8.2 (2.0)			—		—			—		—		—	
	Postintervention time point	7.3 (2.3)		–0.19		7.8 (1.9)			–0.21		0.40 (95)			44.30 (2, 94)		0.31		.69	
	4-month follow-up	7.2 (1.9)		–0.19		7.4 (2.6)			–0.22		0.02 (68)			4.75 (2, 67)		0.06		.98	

^a^Cohen *d* represents the effect sizes of within-group comparisons (with baseline score as the reference) performed using paired *t* tests (2-tailed). *t* test and *P* values are for group comparisons performed using analysis of covariance. In the Somzz and SHE groups, 69% (34/49) and 76% (37/49) of the participants, respectively, consented to take part in the 4-month follow-up.

^b^ANCOVA: analysis of covariance.

^c^ESS: Epworth Sleepiness Scale.

^d^Not applicable.

^e^*P*<.01.

^f^DBAS: Dysfunctional Beliefs and Attitudes About Sleep Scale.

^g^*P*<.001.

^h^PHQ-9: Patient Health Questionnaire–9.

^i^*P*<.05.

^j^GAD-7: Generalized Anxiety Disorder Scale–7.

^k^FSS: Fatigue Severity Scale.

^l^SF-36: 36-item Short Form Health Survey.

^m^EQTOTAL: EQ-5D-5L.

### Concurrent Medications

Medications related to sleep were used by 18% (9/49) of participants in the Somzz group and 18% (9/49) of participants in the SHE group (*χ*^2^_1_=0.0; *P*>.99). Specifically, mirtazapine was used by 2% (1/49) of participants in the Somzz group and none in the SHE group, and benzodiazepines were used by 8% (4/49) of participants in the Somzz group and 14% (7/49) of participants in the SHE group. Z-drugs were used by 10% (5/49) of participants in each group. Antihistamines were used by 2% (1/49) of participants in the Somzz group and 12% (6/49) of participants in the SHE group.

### Factors Associated With Remission and Response in the Somzz Group

Binary regression analysis of remission in the Somzz group revealed that higher DBAS and nondrinking were associated with nonremission (DBAS: estimate=0.11, *z*=2.05, and *P*=.04; drinking: estimate=4.39, *z*=2.25, and *P*=.03). In addition, the binary regression model of responders indicated that a younger age (estimate=0.28; *z*=2.07; *P*=.04), absence of medical history (estimate=–8.01; *z*=–2.01; *P*=.045), and shorter TIB (estimate=0.03; *z*=2.11; *P*=.04) at baseline were significantly associated with the treatment response in the Somzz group.

## Discussion

### Principal Findings

In this study, we found that the Somzz group showed significantly greater improvements in ISI scores, ESS scores, sleep efficiency, WASO, and satisfaction after sleep at the postintervention time point compared to the SHE group. The effect on ISI scores persisted at the 4-month follow-up in the Somzz group. In addition, the Somzz group exhibited lower scores in the ESS, DBAS, PHQ-9, FSS, and SF-36 at the postintervention time point than the SHE group. Among the various methods for delivering digital or internet-based CBTi, to our knowledge, this study is the first to investigate effect of an MCBTi on sleep as well as mental health with a multicenter RCT design using an active control. The Somzz group achieved a favorable attrition rate of 12% (6/49), suggesting that Somzz is an efficacious insomnia treatment with high sustainability of use.

The Somzz group demonstrated a decrease in insomnia severity, as measured using the ISI, starting from 2 weeks after the intervention with a gradual decrease until the postintervention visit. The treatment effect was sustained until the 4-month follow-up, with a moderate to large effect size observed from the second week of the intervention to the 4-month follow-up compared to the baseline ISI score. In the Somzz group, basic education was implemented in the first week followed by stimulus control and sleep restriction in the second week. It appears that stimulus control and sleep restriction play more critical roles in improving insomnia, with a greater decrease in ISI score at week 2. Furthermore, CBTi can initially reduce sleep time, resulting in a possible aggravation of insomnia symptoms, which improve with time [32]. The active comparator app of the SHE group, on the other hand, only includes SHE and a sleep diary module. The SHE group also presented with reduced insomnia severity compared to the baseline from intervention week 3 to the postintervention time point, with a smaller effect size than that of the Somzz group. The findings indicate that SHE has some effect but not sufficient to invoke insomnia improvement [10,33,34], which can be reinforced by other components of CBTi, as indicated by a larger effect size in the Somzz group. Moreover, the impact of SHE might have been influenced by the Hawthorne effect [35] and the natural progression of insomnia [36]. Furthermore, participants with mild insomnia (ISI score of ≥8 and <15) may have partially benefited from SHE. The larger effect size of the ISI score decrease observed in the Somzz group indicates a superior treatment effect on insomnia severity compared to that of the SHE group.

Group differences in ISI scores were noted at the postintervention time point, whereas no significant differences in ISI scores were observed between the Somzz and SHE groups at weeks 1, 2, and 4. Cognitive therapy, TIB restriction, and relapse prevention were the components of the Somzz from week 4 to the postintervention time point. Assuming the “additive model” posited by a component network meta-analysis [18] where the combination therapy effect is viewed as a simple sum of effects without interaction, we presume that cognitive therapy and TIB restriction may have been important factors in improving subjective sleep quality in this study, which aligns with the results of the meta-analysis. Given the limitations of cognitive therapy provided in MCBTi compared to face-to-face CBTi, we believed that the significant effect may be due to the consistent TIB restriction over the 6 weeks in this study. In addition, significant differences in sleep efficiency and SOL between the 2 groups were observed at session 2. This suggests that combination of TIB restriction and stimulus control may have significant effects on sleep efficiency and SOL, which is also consistent with the results of the meta-analysis [18]. However, the conclusions should be interpreted meticulously due to the possible complex interactions among the components of CBTi. Further component network meta-analyses encompassing more MCBTi studies would be helpful for more accurate conclusions.

Although insomnia severity remained significantly reduced compared to baseline at the 4-month follow-up, it showed an increasing trend after the intervention in both the Somzz and SHE groups. Group differences in ISI scores were observed at the postintervention time point and at the 1-, 2-, and 3-month follow-ups, but these differences disappeared at the 4-month follow-up. The lack of difference between the 2 groups was also observed in an earlier study in which group differences in ISI scores were observed at the 3-month follow-up but not at the 6-month follow-up [10]. These results suggest that additional booster sessions approximately 3 months after the intervention may be beneficial in sustaining the better treatment effect of MCBTi. However, the ISI score decrease was relatively maintained in the CBTi group in the previous study [10]. An internet-based CBTi program also showed sustained treatment effects on ISI scores, with a notable effect of continuously decreasing ISI scores observed at the 1-year follow-up [33]. That study did not include a relaxation session; instead, the focus was on other components of CBTi, such as sleep restriction, stimulus control, cognitive restructuring, and sleep hygiene in 6 sessions [33]. This suggests that these components, aside from relaxation therapy, play a critical role in maintaining long-term treatment effects. If we had added a session to reinforce the cognitive component [18], thus providing 7 sessions similar to a previous study on CBTi [10], the effects of CBTi may have been better sustained. In addition, our active comparator app included SHE, providing the same content as Somzz. The education had some treatment effect on insomnia severity in the SHE group, which may have obscured the differences in ISI scores between the 2 groups at the 4-month follow-up. In addition, the waxing or waning nature of insomnia disorder may have contributed to a slight insomnia improvement at the 4-month follow-up in the SHE group.

Furthermore, the effectiveness of the Somzz app could have been confounded by the natural progression of insomnia and the Hawthorne effect. A population-based study reported cumulative remission rates for insomnia disorder among good sleepers of 13.4% after 1 month and 14.2% after 3 months [36] compared with our rate of 45% (22/49) at 6 weeks after the intervention. However, because the group classification criteria differed between our study and that previous study, the results should be compared with caution. Another study investigating the natural history of insomnia over 3 years reported that 74% and 46% of the participants had persistent insomnia for 1 and 3 years, respectively [37]. Although the follow-up period in our study was shorter, and considering that insomnia symptoms may wax and wane, it can be inferred that insomnia disorder can persist without treatment. Parallel RCTs including a third group with no intervention would be optimal for excluding any possible impact of the natural progression of insomnia or the Hawthorne effect on the outcomes of Somzz.

Our study found that the Somzz group had better outcomes in sleep efficiency, WASO, and satisfaction related to sleep based on sleep diary measures compared to the SHE group, which aligns with the results of previous studies [10,33]. Daytime sleepiness measured using the ESS was also improved in the Somzz group, with a significant group difference compared to the SHE group at the postintervention time point. The Somzz group also showed treatment effects on depression and quality of life at the postintervention time point, again consistent with the results of previous studies [38-40]. Although the scores on the PHQ-9 and SF-36 were lower than those at the baseline at the 4-month follow-up, the scores showed a worsening trend in the Somzz group from the postintervention time point to the 4-month follow-up. This trend may be correlated with increased insomnia severity after the intervention, and the correlation can be attributed to the well-established bidirectional interaction of insomnia with depression and quality of life [40]. Similar results were noted in a web-based CBTi study, which showed a treatment effect on depression and suicidal ideation at week 6 but not at the 6-month follow-up when measured using the psychiatric symptom frequency scale [38].

In addition, the Somzz group had significantly lower scores on the DBAS than the SHE group, and this improvement was maintained at the 4-month follow-up. Moreover, higher DBAS scores at baseline were a significant predictor of nonremission. Dysfunctional beliefs about sleep are a crucial factor in perpetuating insomnia as they can provoke a fear of losing control over sleep, worry about the negative consequences of insomnia, and feelings of hopelessness and anxiety [23]. This, in turn, could potentially prolong insomnia symptoms. The results suggest that targeting dysfunctional beliefs may be crucial in effectively treating insomnia using CBTi. Furthermore, a shorter total TIB at baseline was a significant predictor of remission, highlighting the importance of sleep restriction through TIB prescription as a crucial intervention for treating insomnia.

Our study had an attrition rate of 12% (6/49) at the postintervention time point among participants randomized to the Somzz group. This shows relatively good compliance compared to other delivery forms of digital CBTi, with an attrition rate of 21.6% (SD 16.9%) [13], and face-to-face CBTi, with a dropout rate of 14% to 40% [41]. This suggests that the use of MCBTi has the potential to decrease the prevalence of insomnia through high compliance with treatment.

### Limitations

There are several limitations to our study. First, we conducted follow-up for 4 months (18 weeks), which is a relatively short time to infer the long-term effects of Somzz. Second, the impact of Somzz might have been modulated by the Hawthorne effect or the natural progression of insomnia. However, we compared Somzz with SHE in an effort to minimize any influence of such effects. A parallel RCT including a third group with no intervention and with a longer follow-up period and larger sample size is needed to validate our results. Finally, we only used subjective measures of sleep in our study. However, subjective sleep measures including sleep diaries are known to be reliable and valid measures of sleep and correlate with objective measures of sleep [42].

### Conclusions

Our study provides strong evidence that MCBTi can effectively treat insomnia by improving sleep-related variables, depression, and social functioning. MCBTi provides a highly accessible, time-efficient treatment option with high compliance that is suitable for industrious society.

## Appendix

Multimedia Appendix 1 Prespecified analysis plan.

Multimedia Appendix 2 Sleep diary outcomes compared between the Somzz and sleep hygiene education groups.

Multimedia Appendix 3 CONSORT-EHEALTH checklist (v1.6.1).

## Abbreviations

CBTi: cognitive behavioral therapy for insomnia
DBAS: Dysfunctional Beliefs and Attitudes About Sleep Scale
ESS: Epworth Sleepiness Scale
FSS: Fatigue Severity Scale
ISI: Insomnia Severity Index
MCBTi: mobile app–based digital cognitive behavioral therapy for insomnia
PHQ-9: Patient Health Questionnaire–9
RCT: randomized controlled trial
SF-36: 36-item Short Form Health Survey
SHE: sleep hygiene education
SOL: sleep onset latency
TIB: time in bed
WASO: wake after sleep onset

## Data Availability

The data sets generated during and analyzed during this study are available from the corresponding author on reasonable request.
