# Supplementary material for: Efficacy of Mobile App–Based Cognitive Behavioral Therapy for Insomnia: Multicenter, Single-Blind Randomized Clinical Trial
Source: J Med Internet Res. 2024 Jul 26;26:e50555. doi: 10.2196/50555 (PMC11316165; doi:10.2196/50555)
Supplement: Multimedia Appendix 1 [file jmir_v26i1e50555_app1.docx]

Statistical Analysis Plan

Protocol version 2.1, Date 01Sep2021

<Primary Efficacy Assessment>

- Descriptive statistics (number of participants, mean, standard deviation, median, minimum, and maximum) of the change in Insomnia Severity Index (ISI) scores from baseline to the endpoint visit are provided for each group. Statistical significance of differences between groups is assessed using ANCOVA, adjusting for baseline ISI scores as a covariate.

<Secondary Efficacy Assessment>

- Descriptive statistics (number of participants, mean, standard deviation, median, minimum, and maximum) of ISI scores at each time point are provided for each group. Statistical significance of differences between groups is assessed using an Independent two-sample t-test (or Wilcoxon rank sum test if normality assumption is violated).

- Descriptive statistics (number of participants, mean, standard deviation, median, minimum, and maximum) of sleep efficiency (SE) change from baseline to the endpoint visit are provided for each group. Statistical significance of differences between groups is assessed using an Independent two-sample t-test (or Wilcoxon rank sum test if normality assumption is violated).

- The pattern of sleep efficiency (SE) changes from baseline to each time point is examined. Descriptive statistics (number of participants, mean, standard deviation, median, minimum, and maximum) of SE at each time point and the change from baseline to each time point are provided for each group. Statistical significance of differences between groups is assessed using an Independent two-sample t-test (or Wilcoxon rank sum test if normality assumption is violated). Additionally, the statistical significance of SE changes over time between the two groups is assessed using repeated measurement ANOVA, followed by mean plot construction.

- Descriptive statistics (number of participants, mean, standard deviation, median, minimum, and maximum) of ISI scores and the change in ISI scores at each time point are provided for each group. Statistical significance of differences between groups is assessed using an Independent two-sample t-test (or Wilcoxon rank sum test if normality assumption is violated). Additionally, the statistical significance of ISI score changes over time between the two groups is assessed using repeated measurement ANOVA.

- Descriptive statistics (number of participants, mean, standard deviation, median, minimum, and maximum) of the change in sleep onset latency (SOL) from baseline to the endpoint visit are provided for each group. Statistical significance of differences between groups is assessed using an Independent two-sample t-test (or Wilcoxon rank sum test if normality assumption is violated).

- Descriptive statistics (number of participants, mean, standard deviation, median, minimum, and maximum) of the change in wake after sleep onset (WASO) from baseline to the endpoint visit are provided for each group. Statistical significance of differences between groups is assessed using an Independent two-sample t-test (or Wilcoxon rank sum test if normality assumption is violated).

- Descriptive statistics (number of participants, mean, standard deviation, median, minimum, and maximum) of the change in total sleep time (TST) from baseline to the endpoint visit are provided for each group. Statistical significance of differences between groups is assessed using an Independent two-sample t-test (or Wilcoxon rank sum test if normality assumption is violated).

- Descriptive statistics (number of participants, mean, standard deviation, median, minimum, and maximum) of the change in the number of days of sleep aid intake from baseline to visit 4 (14 days after device application) and the endpoint visit are provided for each group. Statistical significance of differences between groups is assessed using an Independent two-sample t-test (or Wilcoxon rank sum test if normality assumption is violated).

- Descriptive statistics (number of participants, mean, standard deviation, median, minimum, and maximum) of the change in Dysfunctional beliefs and Attitudes about sleep-16 (DBAS-16) scores from baseline to the endpoint visit are provided for each group. Statistical significance of differences between groups is assessed using an Independent two-sample t-test (or Wilcoxon rank sum test if normality assumption is violated).

- Descriptive statistics (number of participants, mean, standard deviation, median, minimum, and maximum) of the change in Patient Health Questionnaire-9 (PHQ-9) scores from baseline to the endpoint visit are provided for each group. Statistical significance of differences between groups is assessed using an Independent two-sample t-test (or Wilcoxon rank sum test if normality assumption is violated).

- Descriptive statistics (number of participants, mean, standard deviation, median, minimum, and maximum) of the change in Generalized Anxiety Disorder-7 (GAD-7) scores from baseline to the endpoint visit are provided for each group. Statistical significance of differences between groups is assessed using an Independent two-sample t-test (or Wilcoxon rank sum test if normality assumption is violated).

- Descriptive statistics (number of participants, mean, standard deviation, median, minimum, and maximum) of the change in Fatigue Severity Scale (FSS) scores from baseline to the endpoint visit are provided for each group. Statistical significance of differences between groups is assessed using an Independent two-sample t-test (or Wilcoxon rank sum test if normality assumption is violated).

- Descriptive statistics (number of participants, mean, standard deviation, median, minimum, and maximum) of Epworth sleepiness scale (ESS) scores and the change in ESS scores at each time point are provided for each group. Statistical significance of differences between groups is assessed using an Independent two-sample t-test (or Wilcoxon rank sum test if normality assumption is violated). Additionally, the statistical significance of ESS score changes over time between the two groups is assessed using repeated measurement ANOVA.

** Based on the pre-specified statistical analysis plan, we planned to compare the primary and secondary outcomes in each visit adjusting for the baseline. Based on the previous studies [10, 30] we compared the ISI scores at each time point instead of focusing on ISI changes between the baseline and endpoint visits. We employed ANCOVA to compare the sleep-related and mental health-related scales, as well as sleep diary variables, between the two groups, adjusting for baseline severity. Given that linear mixed modeling (LMM) offers greater flexibility in incorporating random effects, and accommodating correlated data [31], compared to repeated measures ANOVA, we chose to assess the group × time interaction using LMM, following previous methodological approaches [33].
