# Supplementary material for: Efficacy of Mobile App–Based Cognitive Behavioral Therapy for Insomnia: Multicenter, Single-Blind Randomized Clinical Trial
Source: J Med Internet Res. 2024 Jul 26;26:e50555. doi: 10.2196/50555 (PMC11316165; doi:10.2196/50555)
Supplement: Multimedia Appendix 2 [file jmir_v26i1e50555_app2.docx]

**Multimedia Appendix 2.** Sleep diary outcomes compared between the Somzz and sleep hygiene education (SHE) groups.^a^

|  | | Somzz (n=47) | | SHE (n=47) | | ANCOVA^b^ statistics | | | |
| --- | --- | --- | --- | --- | --- | --- | --- | --- | --- |
|  | | Mean (SD) | Cohen *d* | Mean (SD) | Cohen *d* | *t* test (*df*) | *F* test (*df*) | η_p_^2^ | *P* value |
|  | | | | | | | | | |
| **Nap (minutes)** | | | | | | | | | |
|  | Baseline | 23.7 (24.4) | —^c^ | 22.5 (28.0) | —^c^ | —^d^ | —^d^ | —^d^ | —^d^ |
|  | Session 2 | 22.2 (34.1) | –0.04 | 24.6 (33.2) | 0.05 | 0.39 (92) | 1.94 (2, 91) | 0.02 | .70 |
|  | Session 3 | 19.9 (26.5) | –0.16 | 26.5 (34.8) | 0.09 | 1.13 (92) | 3.86 (2, 91) | 0.03 | .26 |
|  | Session 4 | 20.7 (27.5) | –0.10 | 18.4 (22.7) | –0.12 | –0.39 (92) | 1.89 (2, 91) | 0.02 | .70 |
|  | Session 5 | 20.2 (29.1) | –0.12 | 11.4 (16.9) | –0.37^e^ | –1.79 (92) | 5.48 (2, 91) | 0.04 | .08 |
|  | Session 6 | 20.7 (27.7) | –0.10 | 17.5 (26.4) | –0.15 | –0.52 (92) | 3.62 (2, 91) | 0.04 | .60 |
| **Total sleep time (minutes)** | | | | | | | | | |
|  | Baseline | 317.1 (74.5) | —^c^ | 326.0 (100.3) | —^c^ | —^d^ | —^d^ | —^d^ | —^d^ |
|  | Session 2 | 341.8 (94.2) | 0.32 | 350.1 (117.5) | 0.36 | 0.03 (92) | 53.33 (2, 91) | 0.37 | .97 |
|  | Session 3 | 342.9 (95.3) | 0.34 | 362.2 (106.3) | 0.71 | 0.86 (92) | 67.42 (2, 91) | 0.42 | .39 |
|  | Session 4 | 338.3 (86.7) | 0.25 | 344.9 (99.4) | 0.33 | 0.02 (92) | 39.99 (2, 91) | 0.30 | .99 |
|  | Session 5 | 346.4 (82.6) | 0.41 | 356.0 (98.7) | 0.36 | 0.26 (92) | 30.69 (2, 91) | 0.25 | .80 |
|  | Session 6 | 355.0 (86.3) | 0.48^e^ | 347.1 (91.1) | 0.33 | –1.01 (92) | 36.72 (2, 91) | 0.29 | .32 |
| **Sleep efficiency (%)** | | | | | | | | | |
|  | Baseline | 65.7 (14.8) | —^c^ | 64.9 (15.5) | —^c^ | —^d^ | —^d^ | —^d^ | —^d^ |
|  | Session 2 | 74.5 (15.1) | 0.82^f^ | 67.8 (16.9) | 0.27 | –2.87 (92) | 71.04 (2, 91) | 0.42 | .005 |
|  | Session 3 | 75.2 (16.0) | 0.80^f^ | 69.8 (15.8) | 0.48 | –2.19 (92) | 60.55 (2, 91) | 0.39 | .03 |
|  | Session 4 | 76.4 (14.2) | 0.91^g^ | 69.4 (16.8) | 0.51 | –3.08 (92) | 71.92 (2, 91) | 0.42 | .003 |
|  | Session 5 | 76.6 (15.9) | 1.06^g^ | 70.4 (16.2) | 0.56 | –2.76 (92) | 82.57 (2, 91) | 0.46 | .007 |
|  | Session 6 | 78.3 (15.3) | 1.16^g^ | 70.6 (16.7) | 0.53 | –3.32 (92) | 69.87 (2, 91) | 0.41 | .001 |
| **Sleep onset latency (minutes)** | | | | | | | | | |
|  | Baseline | 82.3 (62.0) | —^c^ | 97.1 (78.1) | —^c^ | —^d^ | —^d^ | —^d^ | —^d^ |
|  | Session 2 | 55.1 (43.4) | –0.57^e^ | 91.3 (72.2) | –0.11 | 3.14 (92) | 55.60 (2, 91) | 0.34 | .002 |
|  | Session 3 | 52.5 (50.6) | –0.54^e^ | 86.9 (77.5) | –0.22 | 2.57 (92) | 53.95 (2, 91) | 0.34 | .01 |
|  | Session 4 | 59.8 (78.2) | –0.44 | 88.1 (84.1) | –0.19 | 1.43 (92) | 80.07 (2, 91) | 0.46 | .16 |
|  | Session 5 | 51.5 (76.9) | –0.57^e^ | 88.5 (86.5) | –0.17 | 2.17 (92) | 71.29 (2, 91) | 0.42 | .03 |
|  | Session 6 | 54.1 (78.0) | –0.62 | 80.3 (68.4) | –0.31 | 1.45 (92) | 66.57 (2, 91) | 0.41 | .15 |
| **Number of awakenings** | | | | | | | | | |
|  | Baseline | 1.5 (0.9) | —^c^ | 1.8 (1.8) | —^c^ | —^d^ | —^d^ | —^d^ | —^d^ |
|  | Session 2 | 1.5 (1.4) | –0.02 | 1.8 (2.8) | –0.002 | –0.30 (92) | 160.40 (2, 91) | 0.64 | .76 |
|  | Session 3 | 1.4 (1.1) | –0.14 | 1.7 (1.8) | –0.16 | 0.11 (92) | 188.10 (2, 91) | 0.67 | .91 |
|  | Session 4 | 1.3 (1.2) | –0.30 | 1.6 (1.7) | –0.29 | 0.68 (92) | 134.20 (2, 91) | 0.59 | .50 |
|  | Session 5 | 1.3 (1.1) | –0.28 | 1.6 (1.8) | –0.27 | 0.49 (92) | 125.00 (2, 91) | 0.58 | .63 |
|  | Session 6 | 1.3 (1.1) | –0.30 | 1.6 (1.9) | –0.42 | 0.51 (92) | 189.00 (2, 91) | 0.67 | .61 |
| **Wake after sleep onset (minutes)** | | | | | | | | | |
|  | Baseline | 89.9 (70.1) | —^c^ | 81.2 (49.0) | —^c^ | —^d^ | —^d^ | —^d^ | —^d^ |
|  | Session 2 | 66.7 (64.8) | –0.41 | 75.0 (56.7) | –0.16 | 1.52 (92) | 38.76 (2, 91) | 0.30 | .13 |
|  | Session 3 | 63.7 (64.4) | –0.45 | 70.3 (48.6) | –0.32 | 1.38 (92) | 37.57 (2, 91) | 0.29 | .17 |
|  | Session 4 | 58.7 (56.1) | –0.55^e^ | 66.5 (47.2) | –0.44 | 1.59 (92) | 37.18 (2, 91) | 0.29 | .12 |
|  | Session 5 | 62.1 (57.3) | –0.56^e^ | 63.6 (40.7) | –0.54 | 0.94 (92) | 50.79 (2, 91) | 0.36 | .35 |
|  | Session 6 | 53.0 (52.4) | –0.79^f^ | 65.3 (41.1) | –0.44 | 2.55 (92) | 51.81 (2, 91) | 0.36 | .01 |
| **Refreshment after sleep** | | | | | | | | | |
|  | Baseline | 2.7 (0.5) | —^c^ | 2.5 (0.6) | —^c^ | —^d^ | —^d^ | —^d^ | —^d^ |
|  | Session 2 | 2.7 (0.6) | 0.07 | 2.6 (0.7) | 0.19 | 0.22 (92) | 56.08 (2, 91) | 0.38 | .83 |
|  | Session 3 | 2.7 (0.6) | 0.09 | 2.7 (0.7) | 0.28 | 0.61 (92) | 43.44 (2, 91) | 0.32 | .54 |
|  | Session 4 | 2.8 (0.5) | 0.18 | 2.6 (0.6) | 0.26 | –0.10 (92) | 52.83 (2, 91) | 0.36 | .92 |
|  | Session 5 | 2.8 (0.5) | 0.38 | 2.7 (0.7) | 0.26 | –0.58 (92) | 39.10 (2, 91) | 0.29 | .56 |
|  | Session 6 | 2.9 (0.5) | 0.62^e^ | 2.7 (0.6) | 0.27 | –1.95 (92) | 40.17 (2, 91) | 0.28 | .05 |
| **Satisfaction after sleep** | | | | | | | | | |
|  | Baseline | 2.6 (0.5) | —^c^ | 2.5 (0.6) | —^c^ | —^d^ | —^d^ | —^d^ | —^d^ |
|  | Session 2 | 2.7 (0.6) | 0.14 | 2.6 (0.7) | 0.23 | 0.19 (92) | 47.70 (2, 91) | 0.34 | .85 |
|  | Session 3 | 2.8 (0.6) | 0.27 | 2.6 (0.6) | 0.26 | –0.36 (92) | 38.00 (2, 91) | 0.29 | .72 |
|  | Session 4 | 2.8 (0.5) | 0.35 | 2.6 (0.7) | 0.26 | –0.78 (92) | 40.91 (2, 91) | 0.30 | .44 |
|  | Session 5 | 2.9 (0.6) | 0.43^e^ | 2.7 (0.7) | 0.36 | –0.82 (92) | 34.35 (2, 91) | 0.27 | .42 |
|  | Session 6 | 2.9 (0.6) | 0.55^f^ | 2.7 (0.6) | 0.26 | –2.05 (92) | 26.63 (2, 91) | 0.20 | .04 |

^a^Cohen *d* represents the effect sizes of within-group comparisons (with baseline score as the reference) performed using paired *t* tests (2-tailed). *t* test and *P* values are for group comparisons performed using an analysis of covariance.

^b^ANCOVA: analysis of covariance.

^c^Not applicable.

^d^Not applicable.

^e^*P*<.05.

^f^*P*<.01.

^g^*P*<.001.
